# Analytical Tools to Improve Optimization Procedures for Lateral Flow Assays

**DOI:** 10.3390/diagnostics7020029

**Published:** 2017-05-28

**Authors:** Helen V. Hsieh, Jeffrey L. Dantzler, Bernhard H. Weigl

**Affiliations:** Intellectual Ventures Laboratory/Global Good, Bellevue, 98007 WA, USA; hhsieh@intven.com (H.V.H.); jdantzler@intven.com (J.L.D.)

**Keywords:** immunochromatography, lateral flow, analytical, enzyme-linked immunosorbent assay (ELISA), dynamic light scattering, surface plasmon resonance, global health, point-of-care

## Abstract

Immunochromatographic or lateral flow assays (LFAs) are inexpensive, easy to use, point-of-care medical diagnostic tests that are found in arenas ranging from a doctor’s office in Manhattan to a rural medical clinic in low resource settings. The simplicity in the LFA itself belies the complex task of optimization required to make the test sensitive, rapid and easy to use. Currently, the manufacturers develop LFAs by empirical optimization of material components (e.g., analytical membranes, conjugate pads and sample pads), biological reagents (e.g., antibodies, blocking reagents and buffers) and the design of delivery geometry. In this paper, we will review conventional optimization and then focus on the latter and outline analytical tools, such as dynamic light scattering and optical biosensors, as well as methods, such as microfluidic flow design and mechanistic models. We are applying these tools to find non-obvious optima of lateral flow assays for improved sensitivity, specificity and manufacturing robustness.

## 1. Introduction

Paper chromatography-based assays originated in the first half of the twentieth century, with paraffin-embedded paper for the elution of pigment mixtures. As new materials for these assays were developed, these paper microfluidic assays were expanded from chemistry tests to point-of-care medical diagnostic tests. Formats for these new tests progressed to include immunochromatography, as well as flow through, dipstick or lateral flow designs. These formats use nitrocellulose rather than paper and immunoreagents to detect the presence of the target molecule. More recently, lateral flow assays (LFAs) have returned to paper microfluidic assays to detect proteins of medical interest [1].

Lateral flow tests were initially commercially developed as pregnancy tests in the 1980s [2]. As the technology has advanced, applications include infectious diseases, cardiovascular disease [3], cancer biomarkers [4], foodborne pathogens [5] and veterinary diagnostics [6]. The major advantages for LFAs include their ease of use, rapid response, robustness and low cost, which make these diagnostic tests particularly valued in low resource settings. These advantages to the end-user are balanced by fundamental challenges to the creation and development of the assay. These challenges include the lack of the independent control of primary and secondary binding reactions, loss of assay components due to incomplete binding, potential for false negatives due to the hook effect or false positives due to non-specific binding (NSB). More recent advances have included analytical readers to improve assay sensitivity, remove reliance on user visual acuity and provide quantitative results [7]. 

## 2. Conventional Lateral Flow Assay (LFA) Development

### 2.1. LFA Components

**Figure 1:**
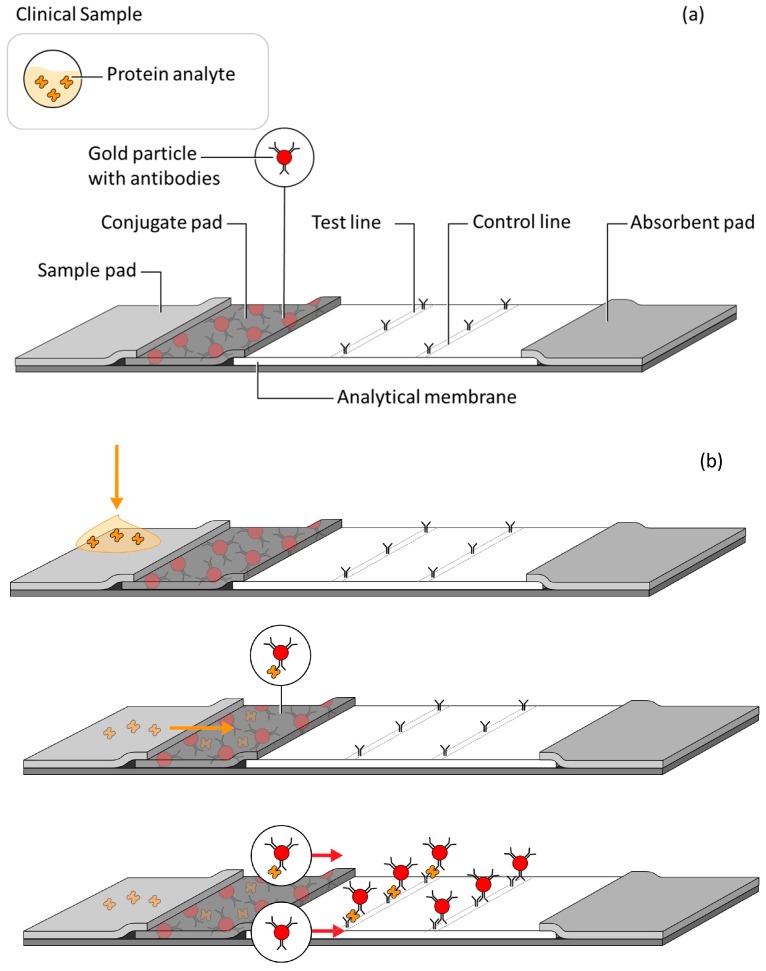
(**a**) Component parts of lateral flow assay strip; (**b**) development of a lateral flow assay test. Yellow arrows indicate the application and flow of the sample; red arrows indicate the flow of the detector nanoparticle.

The typical lateral flow assay test strip is composed of several overlapping membranes (Figure 1a). These usually include a sample pad, a conjugate pad, an analytical membrane (typically nitrocellulose) and an absorption or wicking pad; the biological recognition elements are striped, sprayed or spotted onto the membranes. In this example, the clinical sample containing the protein analyte is applied to the sample pad (Figure 1b, top) and drawn through the additional components by wicking action. The sample, sometimes assisted with a running buffer, travels through the conjugate pad, rehydrating the detector-antibody gold conjugate (Figure 1b, middle), flowing through an analytical membrane striped with a capture antibody (the test line). The formation of the capture antibody-antigen-detector antibody complex is designated as the positive signal (Figure 1b, bottom). Most lateral flow tests include an additional control line, downstream of the test line, to validate proper fluid flow through the test, as well as the activity of the assay reagents.

### 2.2. The Empirical Optimization Process

**Figure 2:**
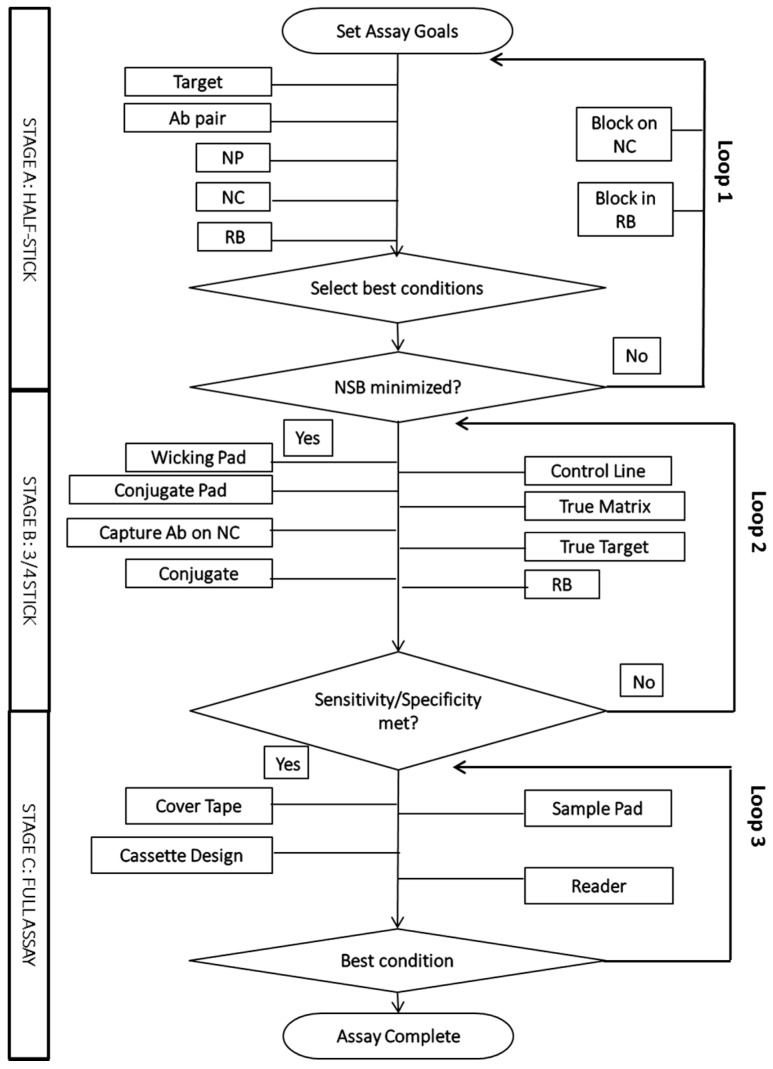
Flowchart indicating typical lateral flow assay (LFA) optimization. Ab, antibody; NP, nanoparticle; NC, nitrocellulose; RB, running buffer.

Development and optimization of an LFA is a complex, iterative process described in Figure 2. A full exploration of the multivariate space can require significant experimentation, labor and time. 

As described in Figure 2, Stage A, the researcher initiates the LFA development by setting assay goals such as initial sensitivity, specificity, sample matrix (type and volume) and time to result. Additional goals may require improvements in comparison to currently available tests or reduction in test costs. After the goals are set, the initial test materials and conditions are identified. Analytical membranes, wicking pads and signal nanoparticles such as colloidal gold, latex beads or cellulose nanobeads can typically be sourced commercially. Biological reagents such as the target antigen, antibodies, biological sample matrices and clinical patient samples, may be difficult to obtain in sufficient quantity for testing; conversely, for some analytes there may be many potential antibody pairs to be tested in the LFA format. Analytical tools described in this review are often used to reduce the number of antibodies to be evaluated, although there is a limit to the predictability of one immunoassay format to another.

For initial testing in LFAs, three components are prepared: the capture antibody test line is striped onto nitrocellulose (NC) membrane, the detector antibody is attached to a nanoparticle (NP) that generates the test signal, and a running buffer (RB) must be selected that allows for the flow of the detector antibody-NP through the nitrocellulose. Here, multiple types of NC and preparations of antibody-NP and RB may be tested to identify the best initial conditions to bring forward. These materials can be quickly screened in the half-stick format, in which the sample and conjugate are applied to the NC-wicking pad in a dipstick format [7]. The best initial conditions with a positive signal and minimal nonspecific binding (NSB) continue directly to Stage B. However, if all conditions tested in Stage A have significant nonspecific binding, a progression through Loop 1 (Figure 2) to consider blocking reagents or a revision of antibody pairs may be required.

When the initial assay screen in Stage A is complete, the LFA may enter Stage B, in which a 3/4 stick format rather than the half stick is used. In Stage B, the antibody-NP is dried on a conjugate pad; this single change requires optimization as the conjugate stability, quantity required and release profile of conjugate upon rehydration can all impact the LFA test parameters. Additional areas of optimization include capture antibody striping conditions, such as antibody concentration and buffer pH, conjugate preparation, the addition of control lines, testing additional wicking pads and the use of true biological targets and matrices rather than model systems. Should these changes reduce the sensitivity or specificity, additional iterations through this area (Loop 2) may be required. NSB or other deleterious effects may be due to antibody-matrix interactions; evaluation in the biological matrix as early as reasonable is encouraged to ensure good performance of a given antibody pair under the anticipated final assay conditions.

When the LFA requirements in Stage B have been met, the assay moves into the final format in Stage C. Additional test components may include a sample pad, cassette housing and/or cover tape. These components may improve assay sensitivity by moderating flow rate and, in some cases, enabling the use of an analytical reader. Here, the LFA most clearly resembles the final commercial product. In addition, an analytical reader may add sensitivity/quantitation, reduce user-based variability and/or allow the application of additional detector molecules (e.g., fluorescent labels.)

While the flowchart in Figure 2 is a typical depiction of LFA development, different components may be pulled earlier into the testing stream or delayed as appropriate. For example, early testing with the true matrix allows for early considerations of sample viscosity, pH and patient to patient variability. However, if supplies of the true matrix are limited in availability, the LFA may be tested with model matrices for initial development, with the understanding that additional optimization may be required when the true matrices are applied.

As described above, there are many interlocking components of a lateral flow test. Many researchers optimize LFAs empirically, searching through a large, multi-variate space. In this review, we will describe the constituents for a typical lateral flow assay and the analytical tools that can be applied to further inform the optimization of these components. Here, we will describe a typical LFA format using a sandwich immunoassay to detect a protein antigen in a clinical sample. Additional LFA formats have been applied to detect non-protein antigens, such as *Mycobacterium tuberculosis* lipoarabinomannan (TB LAM), using a sandwich immunoassay [8], antibodies to infectious diseases using an antigen/anti-human antibody assay [9], or double antigen assay [10], nucleic acids for detection [11] and small molecules, such as using competitive assays [12].

Because of the complexity of traditional empirical LFA optimization, our research group at Intellectual Ventures/Global Good is aiming to improve upon conventional empirical lateral flow assay optimization procedures. We have developed a high-fidelity analytical model to support the design of improved sensitivity lateral flow assays (LFAs) and paper microfluidic assays with LFA-like detection elements [13]. We are applying advanced and in some cases non-standard analytical equipment to monitor and evaluate all aspects of the LFA optimization procedure.

## 3. LFA Components and Analytical Tools for Characterization

The LFA components can be divided into four categories: biological reagents, LFA materials, nanoparticles and the assembled LFA.

### 3.1. Biological Reagents in LFAs

#### 3.1.1. Target Molecules

Identifying and sourcing the LFA target molecule, which may range from proteins, nucleic acids, glycolipids to bacteria, can be surprisingly complex. Identifying the target molecule requires validation of the molecule as a biomarker and attention to the concentration and specificity of the analyte in the clinical sample. Sourcing a target molecule commercially may involve selection from lysate, purified native molecules, recombinant proteins or artificially-produced molecules. It may be preferable to produce the target molecule in-house, requiring additional proficiencies in molecular biology, bacterial or mammalian cell culture, protein expression and purification and/or additional biophysical characterization as described below.

#### 3.1.2. Recognition Molecules 

Antibodies as recognition elements have been a staple of the LFA since its inception. Antibodies may be monoclonal, polyclonal or now produced through antibody engineering methods, such as phage display. While the focus of this review is on direct antigen detection immunoassay LFAs, additional recognition molecules may include other proteins such as antigens for serology tests [10], aptamers [14] or nucleic acids [15].

#### 3.1.3. Clinical Samples

Clinical samples applied to LFAs range from urine in a pregnancy test [16] or a TB LFA [8], plasma or serum for HIV detection [4], whole blood for Dengue fever diagnosis [17] and nasal swabs for flu detection [18]. These varied clinical samples may be quite similar from patient to patient (pH of serum) or quite different for an individual patient, throughout the day (urine). For development of the clinical assay, there is high value in transitioning the assay from a buffer system to the biological matrix quickly. This will allow assay optimization for robustness regarding the vagaries of a clinical sample, such as the wide variability in salt, pH and protein content in urine.

#### 3.1.4. Analytical Tools for Biological Reagents

Analytical tools for the characterization of biological reagents range from basic protein analytical techniques common to many biological laboratories to complex, automated instruments capable of high throughput and high data quality and quantity (Table 1). 

Basic protein analytical techniques common to laboratories may be used to measure the quantity and purity of the protein target and antibodies. UV-Vis spectroscopy and the molecular extinction coefficient can be used to calculate the concentration of purified protein. Protein gel electrophoresis can be used to verify protein size and protein purity [19].

Screening may reduce the list of antibodies to a manageable set prior to development in the LFA itself. The ELISA is a long-established screening tool to identify antibody pairs for possible application in LFA development. Advantages for antibody screening by ELISA include the similarity in the final signaling complex to that of the LFA complex and the high throughput ability to test numerous antibodies pairs, with minimal antibody alteration by the ELISA format. Disadvantages include the time and labor required for ELISAs and potential disparities between the LFA and ELISA formats. The hours of incubation times typical of an ELISA are not representative of the swift resolution (minutes) of a typical LFA. The ELISA is typically governed by a slow kinetic dissociation rate, while the LFA may be dominated by a swift kinetic association rate. Additionally, the ELISA capture antibody and detector antibody designations may be reversed in the LFA. This exchange may be due to differences in antibody modifications in the two assays or may be due to the kinetic parameters described above. The ELISA requires minimal instrumentation, but generally will provide more precise results when automated instrumentation such as a plate washer is employed [20]. A popular, but more costly subset of the ELISA is the Luminex microbead assay. The multiplexing capability of this assay allows rapid identification of capture/detector antibody pairs that can subsequently be incorporated into multiplexed LFAs [21,22].

Optical biosensors based on surface plasmon resonance (SPR) and bio-layer interferometry (BLI) have become popular methods to characterize biological reagents. Biosensors measure the kinetics and affinity of binding between an immobilized ligand and a soluble analyte. These data can be leveraged to determine specific kinetic rate constants of the antibody-target interaction, screen for antibody pairs capable of making a sandwich with the target molecule and determine the fraction of active (i.e., binding competent) protein in a given lot. Employing these tools can streamline the initial search for a high affinity antibody pair capable of forming a sandwich with the target. Unlike ELISA, the timeframe of the optical biosensor assay can be on the order of 30 min, similar to that of the LFA. Careful measurement and assay design can provide information regarding the stoichiometry of the binding event and the activity of the biological reagents. Disadvantages to optical biosensors include the high cost of the instruments. The lower end models of these instruments have good throughput for testing antibody pairs; however, it is the highest end, and most expensive models, that are needed for throughput similar to ELISA [23].

Complex protein analytical techniques are often used to interrogate the quality, as well as quantitatively characterize the biological reagents. The purity, protein aggregation state and the stability of the antibodies will impact the reproducibility and manufacturability of LFAs. As these techniques require significantly more labor and incur higher instrumentation costs, they may be implemented later in the assay development process, when the final candidates have been identified, to insure assay reproducibility.

Light scattering provides a powerful tool to measure the molar mass, oligomerization state and colloidal stability of protein species in solution. Multi-angle static light scattering, UV and refractive index detectors coupled to analytical size exclusion chromatography (SEC), can quantify the distribution of molecular masses present in an impure sample. Both static and dynamic light scattering are able to measure the second virial coefficient, A2, which informs about the tendency of a protein to either self-associate into higher order oligomers or to preferentially associate with the solvent and remain a monomeric unit [25].

Size-exclusion chromatography-multiangle light scattering (SEC-MALS) couples chromatographic separation of biomolecules based on their Stokes radius (i.e., size) with highly accurate mass determination enabled by measuring the scattered light from these separated species. The oligomeric state, percent aggregation and extent of glycosylation are readily determined, providing important quality measures of a given protein preparation. In general, reagents that have the predicted mass and lack aggregates will provide the best performance in downstream assays, such as an LFA [25].

Analytical techniques for protein stability are quite varied. When a protein begins to unfold, previously buried hydrophobic residues become solvent exposed and can promote self-aggregation or non-specific binding to other molecules. To reduce non-specific binding in an LFA, it is advantageous to choose protein reagents that are stable. Intrinsic protein fluorescence, circular dichroism and differential scanning calorimetry can be used to probe the conformational stability of proteins. Intrinsic protein fluorescence spectroscopy measures the changes in tryptophan and tyrosine emission intensity that occur when the local electronic environment changes from buried (folded) to solvent exposed (unfolded). Circular dichroism spectroscopy monitors the loss of chirality as protein secondary structure (α helix & β sheet) is lost upon unfolding. Differential scanning calorimetry measures the change in heat capacity that occurs when a protein transitions from a folded to unfolded state. The denaturation midpoint (T_m_ for thermal and C_m_ for chemical denaturation) between the folded and unfolded states has some predictive value for the conformational stability of a protein in solution. The temperature of unfolding onset and the kinetics of unfolding at elevated temperature can provide additional stability information to guide reagent selection [19]. 

#### 3.1.5. Reference Assays for Clinical Samples

Clinical samples can be interrogated for general sample characteristics, as well as target analyte concentration. In general, lateral flow assay projects, once they have progressed beyond the “spiked analyte in buffer” stage, are further optimized using clinical samples, known to be positive or negative for the analyte to be tested. All clinical samples need to be tested with a reference method to confirm the presence and ideally the concentration of the target analyte. In an ideal case, the reference method tests for the same analyte as the target analyte of the test to be developed and uses an orthogonal method that is more sensitive and less impacted by interference factors. This is not always possible; therefore, PCR assays or culture results for infectious diseases are often used as reference assays for immunoassay development using antigen markers for the same diseases.

ELISAs and Luminex assays described above may have sufficiently high sensitivity to act as reference assays for LFA prototype tests, but as LFAs become more optimized, for example applying high sensitivity detector particles, they can often match ELISA sensitivity [28,29]. The recently developed digital ELISA format employed in the Quanterix Simoa, in which single enzyme-linked immunocomplexes on magnetic beads are detected, shows high promise as a reference assay with exquisite sensitivity [26].

Various clinical chemistry methods and instruments can be employed to improve the understanding of biological matrices or clinical samples to be tested in the LFA [27]. A wide range in some characteristics of the clinical sample, such as pH, salt content, protein content and viscosity, can have significant impact on the sensitivity and specify of the LFA or its susceptibility to confounders, although the need for an explicit measurement may vary. For example, the pH of urine may range from 4.5–8, although it is typically 5.5–6.5 [30]. However, the pH of blood and blood components such as plasma and serum is typically in a quite narrow range [31].

In some cases, clinical samples may be tested for known interferences, such as human anti-mouse antibody (HAMA) or rheumatoid factor [32] to determine if a prototype assay result is due to analyte response, or a response to an interference factor, a non-specific response, or a combination of all of the above. Recent reviews describe the many automated instruments available for the different clinical sample types [27], although point-of-care assays such as dipstick assays can quickly provide semi-quantitative information regarding the clinical samples [30].

### 3.2. LFA Materials 

#### 3.2.1. Membranes and Pads

The LFA platform uses membranes and pads as described above. As many of these materials are commercially available, the manufacturers will have their materials characterized, particularly with respect to flow and adsorbent characteristics, thickness and density. 

#### 3.2.2. Analytical Tools for Characterization of LFA Materials

**Figure 3:**
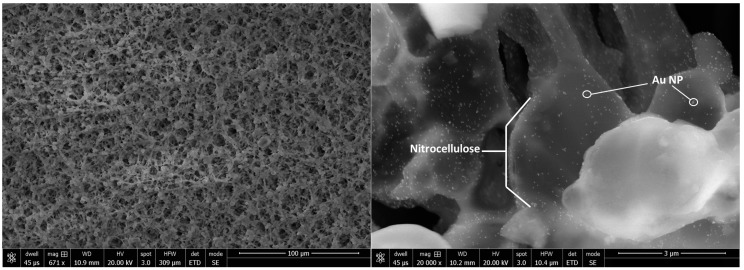
Scanning electron microscopy (SEM) of gold nanoparticles bound to the test line. At 671× magnification (left), the nominal 10-µm pore structure of the nitrocellulose membrane is evident; at 20,000× magnification (right), 40-nm gold nanoparticles are clearly visible. Courtesy of Kevin Nichols, Intellectual Ventures Laboratory.

Scanning electron microscopy (SEM) reconstructs an image at high magnification from the electrons scattered by a focused beam rastering across a sample in a vacuum [33]. This technique can be used to visualize the structure of LFA membranes and pads, as well as the local distribution of gold nanoparticles within a developed LFA (Figure 3).

The flow rate of nitrocellulose membranes is a function of pore size and is reported as capillary rise (s/cm), referring to the time required for water to vertically traverse 4 cm [34]. For commercially available membranes, this information is generally provided by the vendor.

### 3.3. Nanoparticles

#### 3.3.1. Conjugated and Unconjugated Nanoparticles

The detector complex in an LFA is typically the detector antibody passively adsorbed to colloidal gold nanoparticles. While this deector configuration is a staple of many commercial LFA tests, the optimal preparation conditions vary with each antibody and may vary from colloidal gold vendor to vendor. Other detector particles include up-converting phosphors [35], fluorescent molecules [9] or other nanoparticles, such as cellulose nanobeads [18], latex beads [36] or paramagnetic beads [37]. The analytical tools described below (Table 2) can be applied to both conjugated and unconjugated nanoparticles and used to optimize different conjugation strategies. 

#### 3.3.2. Analytical Tools for Characterization of Nanoparticles 

The history of gold NPs [46] and their optical properties are well known and well reviewed [38]. Gold NPs have strong absorption, depending on both the size and shape of the gold particle. The concentration of gold nanoparticles is typically measured by ultraviolet-visible (UV-Vis) spectroscopy. Colloidal gold that is 40 nm in diameter typically has an adsorption peak at 520 nm. The adsorption peaks of larger particles shift towards the red. Gold NPs of different shapes or compositions, such as nanorods or nanoshells, are considerably red shifted from the basic reported diameter. The spectral peak of the gold NP informs about the stability of the gold particle, as aggregated particles will red shift from the expected adsorption peak and broaden the spectral shape.

**Figure 4:**
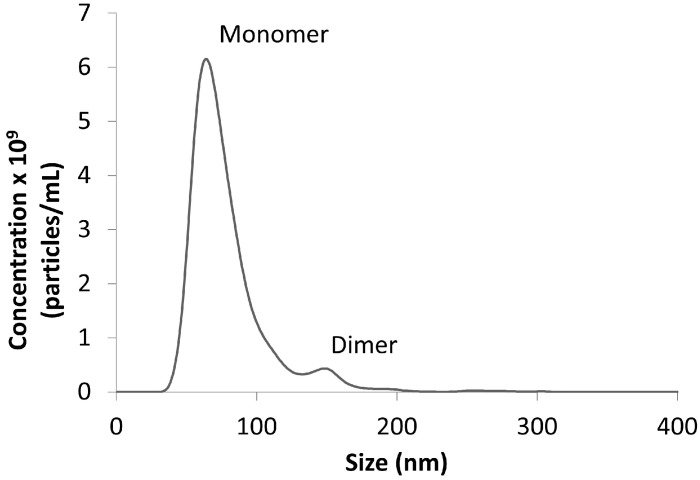
Nanoparticle tracking analysis (NTA) distribution of an antibody-conjugated gold nanoparticle preparation, which is approximately 90% monomeric/10% dimeric.

Dynamic light scattering (DLS) and nanoparticle tracking analysis (NTA) measure the translational diffusion coefficient (D_t_) of particles in solution; this value is related to size and shape. The increase in hydrodynamic radius that occurs during nanoparticle conjugation can be monitored to determine the protein concentration and time required for complete conjugation to occur [40]. These complementary methods are exquisitely sensitive to the presence of larger species, such as aggregated nanoparticles, and can be used to evaluate different conjugation approaches (e.g., buffer conditions) to minimize particle aggregation. DLS is an ensemble average with a wide dynamic size range (0.3 nm–10 µm) that can be applicable to proteins, as well as nanoparticles. NTA measures individual particles, has a more narrow dynamic range (30–1000 nm), a more precise size resolution, and can measure the concentration of nanoparticles [42] (Figure 4). Both DLS and NTA are orders of magnitude more sensitive than UV-Vis [41].

Zeta potential informs about the net charge found on a nanoparticle surface, and it is measured by observing the effect of an applied electric field on particle diffusion (e.g., by using DLS to monitor diffusion). Particles with a high zeta potential tend to repel each other and resist aggregation. The zeta potential of conjugates depends on the composition of the protein hard corona, and the kinetics of corona formation may be monitored by tracking this property over time [44].

Optical biosensors, such as SPR and BLI (described in more detail in Section 3.1.4.), can be used to confirm the activity of conjugated nanoparticles [39]. Once a well-performing particle is identified in an optical biosensor, the LFA materials and running buffer can be optimized to allow flow of this particle in the LFA architecture. 

Transmission electron microscopy (TEM) enables visualization of individual gold nanoparticles. With image analysis software (e.g., ImageJ), the distribution of particle size and shape can be quantified, but characterization of the lower density hard protein corona is difficult [43].

Recently, Worsley et al. [45] employed differential centrifugal sedimentation (DCS) to study the effect of conjugation conditions on particle oligomerization state and the makeup of the protein corona on polystyrene nanoparticles. Conjugates generated in this study were evaluated for LFA signal intensity; high signal correlated with higher antibody mass bound per particle, lower monomer fraction and higher polydispersity [45].

### 3.4. The Assembled LFA 

While the analytical tools described above can assist in selecting and optimizing the preferred assay components, the final assembly of the test is the true arbiter of the successful test.

#### 3.4.1. Visual Scoring

LFAs were originally evaluated by visual scoring, in which the visual presence of a test line indicates the presence of the antigen. While only semi-quantitative, this is still the most direct method to downselect quickly to the most promising conditions and reduce the optimization parameters.

#### 3.4.2. Analytical Readers

Analytical readers can be used to quantitate improvements that may not be obvious with qualitative visual scoring. The LFA visual image may be digitized and analyzed with commercial instruments and software. Imaging systems (Scannex) analyze the whole test and control lines, while scanning instruments (Qiagen, LRE) will record only part of the test lines. The latter instruments thus require more uniform test lines for well-behaved, quantitative results [7]. Thermal contrast readers can be applied to standard gold nanoparticle-based LFAs to improve assay sensitivity. The measurement of a thermal increase generated by laser excitation of gold nanoparticles has been used to increase sensitivity eight-fold in three commercial LFA tests [47]. Additional scanning systems have been developed to detect nanoparticles other than the ubiquitous colloidal gold. A fluorescence LFA reader can be obtained from Qiagen; Wang et al. detected *Bacillus anthracis* spores using a magnetic assay reader from MagnaBiosciences [37]. Camera phone readers have become a popular instrument for developing readers applicable for the developing world [11,48,49].

## 4. Advanced Analytical Optimization Tools

Design of experiments (DOE) is a statistically-based tool that can be used to efficiently screen or optimize multivariate parameter space. This technique has been applied to various technical areas, including pharmaceutical manufacturing [50] and immunoassay development [51]. We are developing an array-based system to incorporate DOE into LFA optimization.

In LFAs, receptor-ligand binding interactions are further confounded by parameters including, but not limited to, capillary flow, diffusion and the rehydration of dried reagents. Mathematical models have been described in the literature to optimize the LFA development strategically, rather than empirically. These models have been used to predict a diverse array of LFA features, ranging from the linearity or non-linearity of signal response [52], the optimal test line placement [53] and the time-dependence of analyte binding [54].

We have developed and validated a mechanistic model to improve LFA and microfluidic assay sensitivity [13]. This model takes input parameters, such as kinetic constants of all reactants, fluidic parameters (e.g., dimensions, porosity, viscosity) and surface energy of the LFA structural materials, as well as optical properties of the signal particles, and tracks the reactions and flow of all constituents through the LFA. By varying the input parameters, the assay can be optimized for sensitivity and/or reagent use within the model; the optimal sensitivity for a given analyte can be predicted. This model has been applied to sequential and batch delivery formats in serology and infectious disease LFAs. Understanding the relationship between antibody-antigen solution kinetic parameters and the more complex LFA assay, in which antibodies are immobilized to nitrocellulose or nanoparticles, will further improve our LFA optimization capabilities at Intellectual Ventures Laboratory/Global Good.

Microfluidic flow design has been used to control the flow and application of sample and reagents [1,55] and to develop multiplex LFAs [56]. In one study, sequential delivery of reagents was found both empirically and by modeling to have greater sensitivity than a premix delivery format [57]. Application of delays, wash buffers and timing manipulation of the clinical sample and/or the nanoparticle can further optimize the LFA. Ideally, these designs and manipulations would be engineered into the LFA cassette to minimize complexity for the end user and retain the high value simplicity of the LFA format.

## 5. Conclusions

Lateral flow assays, while enormously successful in their niche markets, have long been considered “cheap solutions for easy analytical problems”. Several groups are now working to make LFAs substantially more sensitive by adding paper microfluidic features that allow for additional wash or signal amplification steps. While these approaches are very promising, there remains much room for improvement in the realm of traditional low cost LFA technologies. There are many excellent reviews and texts that describe building and optimizing lateral flow assays [3,7,58], but few articles have delved into the analytical toolbox. Using optimization methods and tools such as the ones described in this review may improve the analytical sensitivity and robustness of the LFA, with the potential for better patient outcomes.

## Figures and Tables

**Table 1:** Analytical tools for the characterization of biological reagents. SPR, surface plasmon resonance; BLI, bio-layer interferometry; SEC-MALS, size-exclusion chromatography-multiangle light scattering; DLS, dynamic light scattering; CD, circular dichroism; DCS, differential scanning calorimetry.

Analytical Tool	Applications	Refs
UV-Vis Spectroscopy	Biological reagent concentration	[19]
Gel Electrophoresis	Biological reagent purity	[19]
ELISA	Screening antibody pairs; testing antibody sensitivity/specificity; reference assay	[20]
Luminex	Testing antibody sensitivity/specificity; multiplex systems; reference assay	[21,22]
Optical Biosensors (SPR, BLI)	Screening antibody pairs; antibody sensitivity/specificity; kinetic rates; reagent activity	[23]
SEC-MALS	Biological reagent purity/aggregation state	[24]
DLS	Biological reagent aggregation state; stability	[25]
CD, DSC, intrinsic protein fluorescence	Biological reagent stability	[19]
Digital ELISA	Reference assay	[26]
Automated clinical analyzers	Analysis of clinical samples; reference assay	[27]

**Table 2:** Analytical tools for the characterization of nanoparticles. UV-Vis, ultraviolet-visible; TEM, transmission electron microscopy; DCS, differential centrifugal sedimentation.

Analytical Tool	Applications	References
UV-Vis	Protein adsorption, NP particle concentration/aggregation	[38]
Optical biosensors	Activity	[39]
DLS	Protein adsorption, NP aggregation	[40,41]
Nanoparticle tracking analysis	Protein adsorption, NP concentration/aggregation state	[40,41,42]
TEM	Shape/size	[43]
Zeta potential	Protein adsorption, NP stability	[44]
DCS	Protein adsorption	[45]
